# Are Caregivers’ Feeding Competence and Autonomy Associated with Healthier Restaurant Food Purchases for Their Child at Fast Food or Counter Service Restaurants? A Cross-Sectional Study in a Diverse Sample of U.S. Caregivers

**DOI:** 10.3390/nu16040479

**Published:** 2024-02-07

**Authors:** Violeta Chacón, Sara C. Folta, Erin Hennessy, Tashara M. Leak, Hannah Macfarlane, Christina A. Roberto, Alison Tovar, Norbert Wilson, Christina D. Economos

**Affiliations:** 1 ChildObesity180, Friedman School of Nutrition Science and Policy, Tufts University, 150 Harrison Ave, Boston, MA 02111, USA; 2 Division of Nutritional Sciences, Cornell University, Reservoir Ave, Ithaca, NY 14850, USA; 3 Division of General Internal Medicine, Weill Cornell Medicine, 530 East 70th Street, M-522, New York, NY 10021, USA; 4 Department of Medical Ethics & Health Policy, Perelman School of Medicine, University of Pennsylvania, 423 Guardian Drive Blockley Hall, Philadelphia, PA 19104-4884, USA; 5 Department of Behavioral and Social Sciences, Center for Health Promotion and Health Equity, Brown School of Public Health, Brown University, Providence, RI 02912, USA; 6 Duke Divinity School, Duke University, Durham, NC 27708, USA; 7 World Food Policy Center, Sandford School of Public Policy, Duke University, Durham, NC 27708, USA

**Keywords:** child feeding, competence, autonomy, Self-Determination Theory

## Abstract

This study examined the cross-sectional relationship between caregivers’ perceived competence and autonomy (as defined by the Self-Determination Theory) and their fast food or counter service restaurant food purchases (side dishes, beverage, and dessert) for their child. A U.S. national convenience sample of caregivers with at least one 3–12-year-old child completed an online survey with questions adapted from the Intrinsic Motivation Inventory that measured perceived competence and autonomy for feeding fruits and vegetables and limiting sugar-sweetened beverages (SSBs) and desserts. The survey included four questions asking about their fast food or counter service restaurant food purchases (side dish, beverage, and dessert). We applied logistic and multinomial logistic regression models to examine the associations between competence or autonomy and restaurant orders. Competence and autonomy were associated with ordering fruits and vegetables as side dishes (OR [95% CI], 1.14 [1.06, 1.24] and 1.09 [1.03, 1.14], respectively). However, higher competence was also associated with ordering desserts at restaurants and higher autonomy was associated with lower odds of ordering water. These findings will inform interventions and programs that aim to support caregivers’ psychological needs, like competence and autonomy, to promote supportive environments and healthier restaurant purchases for their children.

## 1. Introduction

On a given day, 36.3% of U.S. children and adolescents consume fast food, and, on average, 13.8% of their daily calories come from fast food [1]. According to data from the National Health and Nutrition Examination Survey (NHANES), consumption at fast-food and full-serve restaurants is associated with increased sugar-sweetened beverages (SSBs) and sodium- and fat-rich foods consumption [2] and poor diet quality [3].

Mirroring the racial and ethnic disparities in diet quality nationally, it is not surprising to observe these disparities in fast food consumption [1,4]. Among children, data from NHANES showed that non-Hispanic white adolescents consumed fewer daily calories from fast food (14.8%) compared to non-Hispanic Black (21.5%) and Hispanic (18.5%) adolescents [1]. Another examination of NHANES, from 2003–2004 to 2017–2018, showed that the proportions of calories from foods purchased at grocery stores significantly decreased, while the proportions of calories from foods purchased at restaurants increased among non-Hispanic Black and Hispanic children, and these remained stable among non-Hispanic white children [3]. However, an examination of nine NHANES cycles, from 2003 to 2018, found that the proportion of calories consumed at restaurants was higher among non-Hispanic White and Black children compared to Hispanic children [3]. In 2016, a cross-sectional national, non-representative survey in 871 U.S. parents found that they visit about 2.5 restaurants in a given week and about 65% purchase a kid’s meal [4]. Parents of younger children were also significantly more likely to purchase healthier beverages, but not healthier sides. Many factors influence fast-food consumption among children and adolescents, including advertising/marketing, physical and economic access, and caregivers’ individual factors [5,6].

Caregivers’ individual factors play an important role in shaping children’s fast-food consumption. Among these individual factors are the caregivers’ psychological needs. Self-Determination Theory (SDT) [7] proposes that people have psychological needs that are essential for their wellbeing, and if fulfilled, they will enhance their intrinsic motivation (which originates from personal choice, interest, or value) to conduct a specific behavior. Competence and autonomy are two of these needs. Competence is the confidence of being able to conduct a certain behavior, and autonomy involves believing that one’s actions, thoughts, and feelings are self-endorsed and authentic [7]. To help support healthy decisions for their child, caregivers need to feel that they have freedom (autonomy) combined with a feeling of personal effectiveness (competence). These psychological needs increase intrinsic motivation to perform and maintain behavior. Therefore, SDT could help to explain, in part, parent’s feeding decisions, as it explains motivation and essential psychological needs. Only a few studies have examined caregivers’ competence and autonomy in the context of parenting and feeding decisions rather than their own dietary intake. The intrinsic motivation of parents is associated with their child’s fruit and vegetable intake [8]. In addition, maternal and adolescent autonomous motivation is significantly associated with adolescents’ SSB consumption [9]. This suggests that increasing caregivers’ competence and autonomy could improve their children’s eating habits by increasing their intrinsic motivation. However, no studies have examined the relationship between caregiver (or parental) competence and autonomy in relation to fast food or counter service restaurant orders for their child.

This paper aims to examine the cross-sectional relationship between caregivers’ perceived competence and autonomy (as defined by the Self-Determination Theory, SDT) and their fast food or counter service restaurant food purchases (beverage, dessert and side dishes) for their child. We hypothesized that caregivers’ perceived competence and autonomy are significantly associated with orders for their child. These findings will inform interventions and programs to support caregivers’ competence and autonomy to promote supportive environments and healthier purchases within fast food or counter service restaurants for their child.

## 2. Methods

### 2.1. Data Source and Sample

In this cross-sectional study, we conducted an online survey on a national sample of U.S. primary caregivers. This online survey assessed their fast food or counter service restaurant orders (beverage, dessert and side dishes) for their youngest child, between 3–12 years old, and was conducted between 1 February 2020 and 12 May 2020. However, all data collected after 13 March 2020, when COVID-19 was declared a national emergency in the U.S. [10], were excluded, as it was expected that children’s consumption and their caregivers’ restaurant food purchases (beverage, dessert and side dishes) would significantly change. The eligibility criteria for this study included primary caregivers (adults between the ages of 18 and 60 years) of at least one child between the ages of 3 and 12 years (questions from the online survey referred to their youngest) living in the U.S. All study participants were required to answer an online survey in English or Spanish and report their use of social media, which had to be at least a few times per week. Black/African American and/or Hispanic/Latina/o/x parents were the target audience for recruitment. Study participants were recruited through Qualtrics Market Division’s pre-existing national consumer panels. Participants were also recruited through community groups in the Greater Boston and Atlanta areas that worked with Black/African American and/or Hispanic/Latina/o/x parents. 

To assess their eligibility, primary caregivers answered an online screener (see Supplementary Materials section for the complete screener survey) that included five questions: (1) whether they were older than 18 y/o; (2) birthyear; (3) currently in the U.S.; (4) social media use; and (5) relationship with child. The screener also included nine demographic questions: (1) education level; (2) ethnicity; (3) race; (4) zip code; (5) gender; (6) number of children between 3 and 12 y/o; (7) age of youngest child; (8) gender of child; and (9) relationship with child. Those who were eligible were asked for their consent and invited to respond to an online survey (see Supplementary Materials for the complete online survey) that included questions regarding their perceived competence and autonomy, as defined by the SDT, and their restaurant food purchases (beverage, dessert and side dishes) for their youngest child between 3 and 12 years of age. The online screener and survey could be accessed from a computer, smartphone, tablet, or similar device. Participants were provided with a USD 25 e-gift card from a grocery store for completing the survey. This study was approved on 1 November 2019, by the Tufts University Social, Behavioral & Educational Research Institutional Review Board (IRB study # 1908017). 

### 2.2. Dependent Variables

Caregivers’ beverage, side, and dessert usual order at restaurants: The online survey included one question evaluating the frequency of consumption of fast food during the past month, from the National Cancer Institute NHANES Dietary Screener Questionnaire [11] (i.e., “During the past month, how often did your X year-old eat fast food?”). We calculated the average consumption frequency, as it has also been reported in studies that have used the Dietary Screener Questionnaire food items to evaluate consumption frequency [9,12]. 

To assess caregivers’ fast food and counter service restaurant orders (i.e., side, beverage, and dessert) for their youngest child, the online survey included four multiple choice questions, adapted from the Kids Live Well (KLW) program [13], asking whether they ordered an adult or kid’s meal, what they ordered as a side dish (i.e., French fries/potatoes, fresh fruit, apple sauce, or yogurt) and beverage (i.e., water, milk, juice, soda, other), and their frequency of ordering a dessert (i.e., never, occasionally, about half the time, more than half the time, or always) at fast food or counter service restaurants. The Kids Live Well program is a set of guidelines proposed by the National Restaurant Association, along with a Scientific Advisory Board, to help restaurants who voluntarily participate in the program. This program aims to offer healthier menu options and help parents select better menu options for their children. According to the program, to increase the consumption of fruits, vegetables, lean protein, whole grains, and low-fat dairy, while limiting unhealthy fats, sugars, and sodium, side dishes should include at least ½ cup of fruits or vegetables, 1 cup of non-fat yogurt or ½ cup of low-fat dairy, or ½ serving of whole grains. According to this program, the side dish options from the online survey were categorized into sides consistent with this standard (i.e., fruit, apple sauce, or yogurt) or inconsistent with the standard (i.e., fries, other). Kids Live Well also includes a standard for “default beverages” categorized as healthier options, which include water (i.e., water, sparkling water, or flavored water, with no added natural or artificial sweeteners), milk (i.e., flavored or unflavored nonfat or low-fat dairy milk or non-dairy beverage that is nutritionally equivalent to fluid milk in a serving size of 8 oz. or less), and juice (i.e., 100% fruit or vegetable juice, or fruit and/or vegetable juice combined with water or carbonated water, with no added natural or artificial sweeteners, in a serving size of 8 oz. or less). In this case, we categorized caregivers’ responses regarding their beverage orders into (1) water, (2) juice, and (3) soda, coke, or pop. Regarding the frequency of ordering dessert, we categorized caregivers’ reported frequency into never/occasionally or more than occasionally (i.e., half of the time, more than half, and always). While main dishes at fast food restaurants are known to have low nutritional quality and contribute children’s sugar, calorie, and saturated fat consumption, increasing accessibility to healthier side dishes, beverages, or desserts could help to improve nutritional quality. Therefore, we decided to focus on caregivers’ side dish, beverage, and dessert orders, as it is common for fast food restaurants to offer default items that are energy-dense and nutrient-poor [14].

### 2.3. Independent Variables

Caregivers’ perceived competence: Nine perceived competence items were adapted from the Intrinsic Motivation Inventory [15]. Three subscales assessed competence (1–5, 1 = lowest and 5 = highest): perceived competence for (1) feeding fruits and vegetables, (2) limiting SSBs, and (3) limiting desserts. We calculated the subscale scores by averaging across items. Cronbach’s alphas were all over 0.89. 

Caregivers’ perceived autonomy: Perceived autonomy scale included three items, also adapted from the Intrinsic Motivation Inventory [15]: (1) “The food I feed my X year-old is not my own choice”; (2) “When I provide healthy food for my X year-old, it’s because I feel like I have to”; and (3) “I believe I have some choice in what food my X year-old eats”. For these items, Cronbach’s alphas were lower than what is recommended (0.6) [15]. Therefore, we analyzed these items separately rather than as a single scale. The third autonomy item had small variability in this measure, and was therefore excluded from analyses. The remaining items were reverse coded so that higher scores would relate to higher autonomy, to be consistent with the competence scales. Therefore, higher scores represent responses related to more autonomy (1–5, 1 = lowest and 5 = highest). 

Type of motivation: We assessed caregivers’ perceived type of motivation with one question, “The reason I would feed my X year-old a healthy meal is (check all that apply)”. This question was adapted from the Health-Care, Self-Determination Theory Packet [16]. Responses were categorized, according to Deci and Ryan [17], as displaying amotivation when participants responded “I would not—I do not see the point” and as displaying extrinsic motivation when participants responded “because other people say I should” or “because I feel guilty when I don’t”. Responses were categorized as displaying intrinsic motivation when participants responded “because I think it is important to make the effort to do so”, “because I consider it consistent with my values”, or “because I enjoy doing so”. We did not include this variable in the models and it was only included in the descriptive results due to the lack of variability.

Demographic characteristics: The online survey included questions that assessed caregivers’ education, ethnicity, race, zip code, gender, relationship to child, number of children, and age and gender of youngest child. Co-variates that were significantly related to the variables of interest or that have been previously reported in the literature to be related were included in the models [18,19,20]. 

### 2.4. Statistical Analyses

To summarize the demographic characteristics of the sample, caregivers’ competence, autonomy, and restaurant food purchases (beverage, dessert and side dishes) for their child, we used descriptive statistics (e.g., mean, SD, frequency, proportions). We conducted a logistic regression to examine whether caregivers’ competence for feeding fruits and vegetables and autonomy were associated with ordering side dishes that were consistent with KLW standards. A logistic regression model examined the association between caregivers’ competence for limiting dessert and autonomy with their frequency of ordering dessert for their child at restaurants. We conducted a multinomial logistic regression model to examine the association between caregivers’ competence for limiting SSBs and autonomy with ordering water, juice or milk, or soda at restaurants. All models were adjusted for caregivers’ age, education, race/ethnicity, number of children, and child’s gender and age. We established significance at the 95% level. 

## 3. Results

Participant characteristics are summarized in Table 1. Our study sample (*n* = 2694) consisted of caregivers mostly older than 30 years (75.7%), with a relationship reported as parent (93.5%). Most caregivers (75.9%) identified as Hispanic/Latino/a/x or Black/African American and most preferred to answer the survey in English (98.0%), rather than Spanish. Over three-fourths (77.4%) of the sample had at least some college education. More than half (53.5%) had only one child and most (46.7%) had a child between 3 and 5 years old.

Table 2 shows the results from the logistic and multinomial regression models. The odds of ordering a meal side consistent with the KLW standard significantly increased as their competence for feeding fruits and vegetables increased (OR [95% CI], 1.14 [1.06, 1.24]). Greater perceived autonomy based on the question “feeding is their own choice” was significantly associated with higher odds of ordering a meal side consistent with the KLW standard (1.09 [1.03, 1.14]). The odds of ordering a dessert “never” or “occasionally” increased as their perceived autonomy “to feed healthy food” (1.22 [1.14, 1.29]) or that “feeding is their own choice” (1.23 [1.17, 1.29]) increased. On the contrary, we found that as caregivers’ competence for limiting desserts only for special occasions increased, the odds of never or occasionally ordering a dessert significantly decreased (0.79 [0.73, 0.86]). Finally, the odds of ordering water significantly increased as caregivers’ competence for limiting SSBs increased (1.41 [1.10, 1.79]), and greater autonomy “to feed healthy food” to their child was associated with lower odds of ordering water (0.74 [0.62, 0.89]) and juice (0.78 [0.65, 0.93]).

## 4. Discussion

The aim of this study was to examine the association between caregivers’ perceived competence and autonomy (as defined by the SDT) and their side, beverage, and dessert orders for their child when they visit fast food or counter service restaurants. We found that higher caregivers’ competences for feeding fruits and vegetables and limiting SSBs are associated with healthier side and beverage orders. However, higher competence for limiting desserts is associated with lower odds of limiting desserts for their child. We also found that higher autonomy seems to help caregivers to choose healthier sides and limit desserts but not choose healthier beverages. This suggests that competence and autonomy could help caregivers to make healthier feeding decisions at restaurants, but at the same time, other factors might be shaping their restaurant food purchases (beverage, dessert and side dishes) for their child.

Parenting and child feeding are complex processes that are influenced by several factors, including individual and environmental, shape caregivers’ orders at restaurants [5,6]. Caregivers’ competence could be one of many individual factors that explain caregivers’ restaurant orders for their child. Our study suggests that caregivers with higher competence for feeding fruits and vegetables make healthier restaurant food purchases (beverage, dessert and side dishes) for their child. According to the SDT, competence enhances a person’s intrinsic motivation, leading to more persistent behaviors, and feelings of wellbeing [21]. Intrinsic motivation has been associated with caregivers’ own fruit, vegetable, and SSB intake and that of their children [8,9]. Our results suggest that caregivers’ competence translates outside the home when they decide what to order their child at restaurants. However, we also found that as caregivers’ competence for limiting desserts only for special occasions increased, the odds of never or occasionally ordering dessert significantly decreased. This suggests that a caregiver that feels competent may also appreciate the role of a treat, in this case a dessert, as a good way to avoid overeating or fostering competence in their child by teaching them to decide when, how often, and how much dessert to have [22,23]. 

One important environmental factor that shapes caregivers’ beverage orders at fast food restaurants is the availability of SSBs at restaurants [24,25]. Fast food has been linked to SSB consumption among adolescents [26]. SSBs are usually the default beverage offered in children’s menus at fast food restaurants. A recent evaluation of 11-year trends in the availability of children’s meals found that, even though the healthier beverage options have increased, only 20% of meal bundles included default sides or beverages that were considered healthy [14]. Another study in 2019 examining children’s menus at fast food and full-service restaurants found that SSBs constituted the largest percentage of beverages offered (79−81%) [27]. Another examination of the children’s meals at the 50 largest chain restaurants by revenue in the U.S. found that 71.9% failed to meet nutrition standards derived from key nutrition recommendations in the Dietary Guidelines for Americans [28]. However, this study also found that the largest restaurant chains, by number of outlets, have improved their nutrition standards, suggesting that they are capable of changing their meal offerings to make healthier meals for children [28]. Despite the potentially strong environmental influence at restaurants encouraging caregivers to order SSBs, we found that caregivers’ competence for limiting SSBs is significantly associated with their reports on ordering healthier beverage orders, like water, for their child. This result is consistent with a cross-sectional study conducted among Swedish adults that found that higher competence, as defined by SDT, was associated with a healthier dietary pattern, that included lower consumption of SSBs [29].

Another important finding from our study was that higher autonomy, specifically feelings that the food they feed their child is their own choice, is associated with higher odds of ordering a side aligned with the KLW standard and of ordering a dessert never or occasionally. This suggests that caregivers who feel like they have a sense of freedom over their restaurant food purchases might be more likely to order healthier sides and order dessert less frequently. However, we also found that higher autonomy for “feeding healthy food” was associated with lower odds of ordering water and juice, which are considered healthier beverage choices. Although this finding was unexpected, it is possible that parents are influenced by their environment, which is not supportive of their autonomy. Restaurant advertising can influence caregivers’ evaluation of foods and beverages to motivate them to buy their products [30,31]. This could contribute to frustrating caregivers’ autonomy, especially those who are disproportionately targeted by fast food advertising, like Black/African American and/or Hispanic/Latina/o/x caregivers [32], who also face greater risks of obesity and diet-related diseases [1,2]. It is also possible that caregivers might have compensatory behaviors, by making healthy food choices and allowing a less healthy drink.

This study has its limitations. First, caregivers reported their usual restaurant orders for their child, which could introduce social desirability bias. Second, our online screener and survey may not have reached caregivers without Internet access or those who were not technologically savvy. Third, this was a cross-sectional study, which prevented us from establishing a causal relationship between caregivers’ competence and autonomy and their fast-food restaurant orders. Fourth, we did not evaluate other factors that could have an important influence on caregivers’ restaurant orders and their child’s consumption, like caregivers’ and child’s body weight or caregivers’ feeding style. However, this study also has its strengths. Black/African American and Hispanic/Latina/o/x caregivers living in the U.S responded to competence and autonomy questions that were specific to child feeding, regarding which little has been reported. In addition, this study evaluates the associations between SDT constructs (competence and autonomy) and their fast food restaurant orders. Currently, few studies have explored these associations. Finally, SDT is an appropriate framework for explaining the role played by caregivers in their child’s eating habits. These findings help to understand caregiver and child behaviors that can inform future interventions to encourage healthier fast food restaurant orders. 

This study provides important insight into the role that caregivers’ competence and autonomy play when they visit restaurants with their child. Our results suggest that interventions that increase caregivers’ competence and autonomy when ordering at restaurants could promote healthier food purchases (beverage, dessert and side dishes). At the same time, it is important to address the environmental factors that could be undermining caregivers’ ability to make free and autonomous choices when eating out at restaurants. Future research could help to address other important research questions related to the role of caregivers’ competence and autonomy when ordering at restaurants. This could include examining how social, economic, and structural factors influence caregivers’ choices and skills. Exploring children’s competence and autonomy can also help us to understand how they make decisions at restaurants.

## Figures and Tables

**Table 1:** Sociodemographic characteristics of caregivers and their youngest child (*n* = 2694) ^a^.

	N	%
Caregivers’ age ^b^		
19–30	656	24.4
31–40	1314	48.8
>40	724	26.9
Relationship		
Parent	2515	93.5
Other caregiver ^c^	176	6.5
Preferred language		
English	2640	98.0
Spanish	54	2.0
Caregivers’ race/ethnicity		
Hispanic/Latino/a/x, all race	1021	38.0
Non-Hispanic/Latino/a/x, Black	1020	37.9
Other than Not Hispanic/Latin/a/x or Black	644	23.9
Caregiver’s Education level		
High school or less	607	22.6
Some college education/Associate degree	1027	38.2
Complete college	694	25.8
Graduate degree	361	13.4
Number of children		
One	1440	53.5
Two or more	1254	46.5
Age of youngest child ^b^		
3–5	1257	46.7
6–9	894	33.2
10–12	543	20.2
Caregivers’ psychosocial constructs scores, mean ± SD ^d^		
Perceived competence for feeding F & V	4.1 ± 0.98	
Perceived competence for limiting SSBs	4.1 ± 1.02	
Perceived competence for limiting desserts	3.8 ± 1.13	
Perceived autonomy for feeding “healthy food” ^e^	2.6 ± 1.42	
Perceived autonomy: “Feeding is my own choice” ^f^	3.5 ± 1.54	
Motivation types, *n* (%)		
Amotivation	0	0.0
Extrinsic	1465	83.2
Intrinsic	297	16.9
Child’s consumption frequency (times/day, mean ± SD) and caregivers’ restaurant orders for child (*n*, %)		
Fruits and vegetables	0.48 ± 0.36	
SSBs	0.33 ± 0.65	
Desserts	0.22 ± 0.22	
Fast-food	0.16 ± 0.22	
Ate fast food in the past month, *n* (%)	2198 (81.6)	
Frequency of ordering		
Fruit, applesauce, or yogurt as side	1694 (62.9)	
Beverage within the Kids Live Well standard	1895 (69.0)	
Dessert (more than occasionally)	341 (15.6)	

^a^ Excluding all observations after 13 March 2020 (when COVID-19 was declared a national emergency in the U.S.). ^b^ Caregivers’ and child’s age were used as continuous variables in the models. ^c^ Grandparent, aunt/uncle, foster parent, sibling. ^d^ Scores range from 1 to 5, with 5 being the highest score. ^e^ Reversed autonomy question: “When I provide healthy food for my X year-old, it’s because I feel like I have to”. ^f^ Reversed autonomy question: “The food I feed my X year-old is not my own choice”.

**Table 2:** Adjusted ^a^ odds ratio (OR) and relative risk ratio (RRR) (95% CI) of caregivers’ side, beverage, and dessert orders when visiting a restaurant with their child associated with their competence and autonomy.

	Ordering a side within the KLW standard			
	OR (95% CI)		*p*	
Competence in feeding fruits and vegetables	1.14 (1.06, 1.24)		0.001	
Perceived autonomy				
For feeding “healthy food” ^b^	1.04 (0.99, 1.11)		0.134	
“Feeding is my own choice” ^c^	1.09 (1.03, 1.14)		0.002	
	Ordering dessert never or occasionally			
	OR (95% CI)		*p*	
Competence for limiting desserts only for special occasions	0.79 (0.73, 0.86)		0.001	
Perceived autonomy				
For feeding “healthy food” ^b^	1.22 (1.14, 1.29)		<0.001	
“Feeding is my own choice” ^c^	1.23 (1.17, 1.29)		<0.001	
	Beverage orders			
	Ordering water ^d^		Ordering juice ^d^	
	RRR (95% CI)	*p*	RRR (95% CI)	*p*
Competence for limiting SSBs	1.41 (1.10, 1.79)	0.006	1.13 (0.90, 1.44)	0.277
Perceived autonomy				
For feeding “healthy food” ^b^	0.74 (0.62, 0.89)	0.002	0.78 (0.65, 0.93)	0.006
“Feeding is my own choice” ^c^	0.84 (0.69, 1.02)	0.074	0.92 (0.76, 1.10)	0.362

^a^ All models were adjusted for caregiver’s age, education, race/ethnicity, number of children and child’s gender and age. ^b^ Reversed autonomy question: “When I provide healthy food for my X year-old, it’s because I feel like I have to”. ^c^ Reversed autonomy question: “The food I feed my X year-old is not my own choice”. ^d^ reference group = caregivers who order soda.

## Data Availability

The authors do not have permission to share data.

## Supplementary Materials

The following supporting information can be downloaded at: https://www.mdpi.com/article/10.3390/nu16040479/s1, Online survey.
