# Supplementary material for: Are Caregivers’ Feeding Competence and Autonomy Associated with Healthier Restaurant Food Purchases for Their Child at Fast Food or Counter Service Restaurants? A Cross-Sectional Study in a Diverse Sample of U.S. Caregivers
Source: Nutrients. 2024 Feb 7;16(4):479. doi: 10.3390/nu16040479 (PMC10892913; doi:10.3390/nu16040479)
Supplement: Supplementary file 1 [file nutrients-16-00479-s001.zip › nutrients-2825597-supplementary.pdf]

Supplemental file
Survey:

Please answer the following questions:

1. Are you 18 or older?
   - ☐ Yes
   - ☐ No

2. What year were you born? ___________

3. Are you currently in the United States?
   - ☐ Yes
   - ☐ No

4. How often do you use each of the following social networks?

	Daily	A few times a week	Once a week	Rarely	Never
Facebook					
Instagram					
Snapchat					
Twitter					
LinkedIn					
Pinterest					

5. Are you the **primary caregiver** for any children between the ages of 3 and 12?
   - ☐ Yes
   - ☐ No

6. What is your highest level of education?
   - ☐ Less than 8th grade
   - ☐ Some high school (grades 9 to 11)
   - ☐ High school graduate (finished 12th grade) or GED
   - ☐ Some college but no degree
   - ☐ Associate’s degree (AA, AS, etc.)
   - ☐ Bachelor’s degree (BA, BS, etc.)
   - ☐ Graduate degree (MA, MS, MPH, JD, PhD, MD, etc.)
   - ☐ Prefer not to answer

7. Do you identify as Hispanic or Latinx?
   - ☐ Yes
   - ☐ No
   - ☐ Prefer not to answer

8. Do you consider yourself to be:
   - ☐ American Indian or Alaska Native
   - ☐ Asian
   - ☐ Black or African American
   - ☐ Native Hawaiian or other Pacific Islander
   - ☐ White
   - ☐ Some other race (specify): _____________________________
   - ☐ Prefer not to answer

9. What is your zip code? __________________

1. What is your gender?
   - ☐ Male
   - ☐ Female
   - ☐ Prefer to self-identify: ___________________
   - ☐ Prefer not to answer

2. How many children do you have between the ages of 3 and 12 years old?
   - ☐ One
   - ☐ Two
   - ☐ Three
   - ☐ Four or more

3. *If one is selected:* How old is that child?
   - ☐ 3
   - ☐ 4
   - ☐ 5
   - ☐ 6
   - ☐ 7
   - ☐ 8
   - ☐ 9
   - ☐ 10
   - ☐ 11
   - ☐ 12

   If two or more is selected: Of your children who are aged 3-12, how old is the **youngest**?

- ☐ 3
- ☐ 4
- ☐ 5
- ☐ 6
- ☐ 7
- ☐ 8
- ☐ 9
- ☐ 10
- ☐ 11
- ☐ 12

4. What is the sex of that child?
   - ☐ Male
   - ☐ Female
   - ☐ Prefer not to answer

5. What is your relationship to that child?
   - ☐ Parent
   - ☐ Foster parent
   - ☐ Grandparent
   - ☐ Sibling
   - ☐ Aunt or uncle
   - ☐ Other ____________________

Please think of your child who is **X** years old when you answer the remaining questions.

For the next set of questions, please rate how much you agree with each statement when it comes to your **X** year-old.

6. I think I'm pretty good at feeding my **X** year-old fruits and vegetables. Choose one.
   - ☐ Strongly agree
   - ☐ Somewhat agree
   - ☐ Neither agree nor disagree
   - ☐ Somewhat disagree
   - ☐ Strongly disagree
   - ☐ Prefer not to answer

7. I am satisfied with my ability to feed my **X** year-old fruits and vegetables. Choose one.
   - ☐ Strongly agree
   - ☐ Somewhat agree
   - ☐ Neither agree nor disagree
   - ☐ Somewhat disagree
   - ☐ Strongly disagree
   - ☐ Prefer not to answer

8. I am skilled at feeding my **X** year-old fruits and vegetables. Choose one.
   - ☐ Strongly agree
   - ☐ Somewhat agree
   - ☐ Neither agree nor disagree
   - ☐ Somewhat disagree
   - ☐ Strongly disagree
   - ☐ Prefer not to answer

9. I think I'm pretty good at saying no when my **X** year-old asks for sugar-sweetened beverages. Choose one.
   - ☐ Strongly agree
   - ☐ Somewhat agree
   - ☐ Neither agree nor disagree
   - ☐ Somewhat disagree
   - ☐ Strongly disagree
   - ☐ Prefer not to answer

10. I am satisfied with my ability to say no when my **X** year-old asks for sugar-sweetened beverages. Choose one.
    - ☐ Strongly agree
    - ☐ Somewhat agree
    - ☐ Neither agree nor disagree
    - ☐ Somewhat disagree
    - ☐ Strongly disagree
    - ☐ Prefer not to answer

11. I am skilled at saying no when my **X** year-old asks for sugar-sweetened beverages. Choose one.
    - ☐ Strongly agree
    - ☐ Somewhat agree
    - ☐ Neither agree nor disagree
    - ☐ Somewhat disagree
    - ☐ Strongly disagree
    - ☐ Prefer not to answer

12. I think I'm pretty good at limiting desserts for my **X** year-old to special occasions only. Choose one.
    - ☐ Strongly agree
    - ☐ Somewhat agree
    - ☐ Neither agree nor disagree
    - ☐ Somewhat disagree
    - ☐ Strongly disagree
    - ☐ Prefer not to answer

13. I am satisfied with my ability to limit desserts for my **X** year-old to special occasions only. Choose one.
    - ☐ Strongly agree
    - ☐ Somewhat agree
    - ☐ Neither agree nor disagree
    - ☐ Somewhat disagree
    - ☐ Strongly disagree
    - ☐ Prefer not to answer

14. I am skilled at limiting desserts for my **X** year-old to special occasions only. Choose one.
    - ☐ Strongly agree
    - ☐ Somewhat agree
    - ☐ Neither agree nor disagree
    - ☐ Somewhat disagree
    - ☐ Strongly disagree
    - ☐ Prefer not to answer

15. I believe I have some choice in what food my **X** year-old eats. Choose one.
    - ☐ Strongly agree
    - ☐ Somewhat agree
    - ☐ Neither agree nor disagree
    - ☐ Somewhat disagree
    - ☐ Strongly disagree
    - ☐ Prefer not to answer

16. The food I feed my **X** year-old is not my own choice. Choose one.
    - ☐ Strongly agree
    - ☐ Somewhat agree
    - ☐ Neither agree nor disagree
    - ☐ Somewhat disagree
    - ☐ Strongly disagree
    - ☐ Prefer not to answer

17. When I provide healthy food for my **X** year-old, it’s because I feel like I have to. Choose one.
    - ☐ Strongly agree
    - ☐ Somewhat agree
    - ☐ Neither agree nor disagree
    - ☐ Somewhat disagree
    - ☐ Strongly disagree
    - ☐ Prefer not to answer

18. I feel close and connected with other moms on social media because of a shared interest in raising healthy kids. Choose one.
    - ☐ Strongly agree

- ☐ Somewhat agree
- ☐ Neither agree nor disagree
- ☐ Somewhat disagree
- ☐ Strongly disagree
- ☐ Prefer not to answer

19. I feel a sense of intimacy with other moms on social media because of a shared interest in raising healthy kids. Choose one.
    - ☐ Strongly agree
    - ☐ Somewhat agree
    - ☐ Neither agree nor disagree
    - ☐ Somewhat disagree
    - ☐ Strongly disagree
    - ☐ Prefer not to answer

20. I feel a sense of contact with other moms on social media because of a shared interest in raising healthy kids. Choose one.
    - ☐ Strongly agree
    - ☐ Somewhat agree
    - ☐ Neither agree nor disagree
    - ☐ Somewhat disagree
    - ☐ Strongly disagree
    - ☐ Prefer not to answer

21. The reason I would feed my **X** year-old a healthy meal is (check all that apply):
    - ☐ I would not – I do not see the point
    - ☐ Because other people say I should
    - ☐ Because I feel guilty when I don't
    - ☐ Because I think it is important to make the effort to do so
    - ☐ Because I consider it consistent with my values
    - ☐ Because I enjoy doing so
    - ☐ Prefer not to answer

For the next few questions, please think about your child's behavior **over the last month**.

22. During the past month, did your **X** year-old drink **water**? Include flavored water, seltzer; do **not** include water with added sweetener.
    - ☐ Yes
    - ☐ No
    - ☐ Don't know
    - ☐ Prefer not to answer.

23. *If yes to 4* During the past month, how often did your **X** year-old drink **water**?
    - ☐ 1 time last month

- ☐ 2-3 times last month
- ☐ 1 time per week
- ☐ 2 times per week
- ☐ 3-4 times per week
- ☐ 5-6 times per week
- ☐ 1 time per day
- ☐ 2-3 times per day
- ☐ 4-5 times per day
- ☐ 6 or more times per day
- ☐ Prefer not to answer

24. During the past month, did your **X** year-old drink **milk**? Include regular milks, chocolate or other flavored milks, lactose-free milk; do **not** include soy milk, almond milk, other non-dairy milks.
    - ☐ Yes
    - ☐ No
    - ☐ Don't know
    - ☐ Prefer not to answer

25. *If yes to 6* During the past month, how often did your **X** year-old drink **milk**?
    - ☐ 1 time last month
    - ☐ 2-3 times last month
    - ☐ 1 time per week
    - ☐ 2 times per week
    - ☐ 3-4 times per week
    - ☐ 5-6 times per week
    - ☐ 1 time per day
    - ☐ 2-3 times per day
    - ☐ 4-5 times per day
    - ☐ 6 or more times per day
    - ☐ Prefer not to answer

26. *If yes to 6* During the past month, what kind of milk did your **X** year-old usually drink? Choose one.
    - ☐ Whole or regular milk
    - ☐ 2% fat or reduced-fat milk
    - ☐ 1%, ½%, or low-fat milk
    - ☐ Fat-free, skim or nonfat milk
    - ☐ Soy milk
    - ☐ Other kind of milk: ___________________________
    - ☐ Prefer not to answer

27. During the past month, did your **X** year-old drink **regular soda or pop** that contains sugar? Do **not** include diet soda.
    - ☐ Yes

☐ No
☐ Don’t know
☐ Prefer not to answer

28. *If yes to 9* During the past month, how often did your **X** year-old drink **regular soda or pop**?
☐ 1 time last month
☐ 2-3 times last month
☐ 1 time per week
☐ 2 times per week
☐ 3-4 times per week
☐ 5-6 times per week
☐ 1 time per day
☐ 2-3 times per day
☐ 4-5 times per day
☐ 6 or more times per day
☐ Prefer not to answer

29. During the past month, did your **X** year-old drink **100% pure fruit juices** such as orange, mango, apple, grape and pineapple juices? Do **not** include fruit-flavored drinks with added sugar or fruit juice you made at home and added sugar to.
☐ Yes
☐ No
☐ Don’t know
☐ Prefer not to answer

30. *If yes to 11* During the past month, how often did your **X** year-old drink **100% pure fruit juices**?
☐ 1 time last month
☐ 2-3 times last month
☐ 1 time per week
☐ 2 times per week
☐ 3-4 times per week
☐ 5-6 times per week
☐ 1 time per day
☐ 2-3 times per day
☐ 4-5 times per day
☐ 6 or more times per day
☐ Prefer not to answer

31. During the past month, did your **X** year-old drink sweetened fruit drinks, sports or energy drinks, such as Kool-Aid, lemonade, Hi-C, cranberry drink, Gatorade, Red Bull, or Vitamin Water? Include fruit juices you made at home and added sugar to. Do **not** include diet drinks or artificially sweetened drinks.
☐ Yes
☐ No

- ☐ Don’t know
- ☐ Prefer not to answer

32. *If yes to 13* During the past month, how often did your **X** year-old drink **sweetened fruit drinks, sports or energy drinks**?
    - ☐ 1 time last month
    - ☐ 2-3 times last month
    - ☐ 1 time per week
    - ☐ 2 times per week
    - ☐ 3-4 times per week
    - ☐ 5-6 times per week
    - ☐ 1 time per day
    - ☐ 2-3 times per day
    - ☐ 4-5 times per day
    - ☐ 6 or more times per day
    - ☐ Prefer not to answer

33. During the past month, did your **X** year-old eat **fruit**? Include fresh, frozen or canned fruit; do **not** include juice.
    - ☐ Yes
    - ☐ No
    - ☐ Don’t know
    - ☐ Prefer not to answer

34. *If yes to 15* During the past month, how often did your **X** year-old eat **fruit**?
    - ☐ 1 time last month
    - ☐ 2-3 times last month
    - ☐ 1 time per week
    - ☐ 2 times per week
    - ☐ 3-4 times per week
    - ☐ 5-6 times per week
    - ☐ 1 time per day
    - ☐ 2 or more times per day
    - ☐ Prefer not to answer

35. During the past month, did your **X** year-old eat a green leafy or lettuce **salad**, with or without other vegetables?
    - ☐ Yes
    - ☐ No
    - ☐ Don’t know
    - ☐ Prefer not to answer

36. *If yes to 17* During the past month, how often did your **X** year-old eat **salad**?
    - ☐ 1 time last month

- ☐ 2-3 times last month
- ☐ 1 time per week
- ☐ 2 times per week
- ☐ 3-4 times per week
- ☐ 5-6 times per week
- ☐ 1 time per day
- ☐ 2 or more times per day
- ☐ Prefer not to answer

37. During the past month, did your **X** year-old eat any kind of **fried potatoes**, including french fries, home fries, or hash brown potatoes?
    - ☐ Yes
    - ☐ No
    - ☐ Don't know
    - ☐ Prefer not to answer

38. *If yes to 19* During the past month, how often did your **X** year-old eat **fried potatoes**?
    - ☐ 1 time last month
    - ☐ 2-3 times last month
    - ☐ 1 time per week
    - ☐ 2 times per week
    - ☐ 3-4 times per week
    - ☐ 5-6 times per week
    - ☐ 1 time per day
    - ☐ 2 or more times per day
    - ☐ Prefer not to answer

39. During the past month, did your **X** year-old eat any **other kind of potatoes**, such as baked, boiled, mashed potatoes, sweet potatoes, or potato salad?
    - ☐ Yes
    - ☐ No
    - ☐ Don't know
    - ☐ Prefer not to answer

40. *If yes to 21* During the past month, how often did your **X** year-old eat any **other kind of potatoes**?
    - ☐ Never
    - ☐ 1 time last month
    - ☐ 2-3 times last month
    - ☐ 1 time per week
    - ☐ 2 times per week
    - ☐ 3-4 times per week
    - ☐ 5-6 times per week
    - ☐ 1 time per day

- ☐ 2 or more times per day
- ☐ Prefer not to answer

41. During the past month, did your **X** year-old eat **refried beans, baked beans, beans in soup, pork and beans or any other type of cooked dried beans**? Do **not** include green beans.
    - ☐ Yes
    - ☐ No
    - ☐ Don't know
    - ☐ Prefer not to answer

42. *If yes to 23* During the past month, how often did your **X** year-old eat **refried beans, baked beans, beans in soup, pork and beans or any other type of cooked dried beans**?.
    - ☐ 1 time last month
    - ☐ 2-3 times last month
    - ☐ 1 time per week
    - ☐ 2 times per week
    - ☐ 3-4 times per week
    - ☐ 5-6 times per week
    - ☐ 1 time per day
    - ☐ 2 or more times per day
    - ☐ Prefer not to answer

43. During the past month, not including what you just told me about (green salads, potatoes, cooked dried beans), did your **X** year-old eat **other vegetables**?
    - ☐ Yes
    - ☐ No
    - ☐ Don't know
    - ☐ Prefer not to answer
44. *If yes to 25* During the past month, how often did your **X** year-old eat **other vegetables**?
    - ☐ 1 time last month
    - ☐ 2-3 times last month
    - ☐ 1 time per week
    - ☐ 2 times per week
    - ☐ 3-4 times per week
    - ☐ 5-6 times per week
    - ☐ 1 time per day
    - ☐ 2 or more times per day
    - ☐ Prefer not to answer

45. During the past month, did your **X** year-old eat **chocolate or any other types of candy**? Do **not** include sugar-free candy.
    - ☐ Yes
    - ☐ No
    - ☐ Don't know

- ☐ Prefer not to answer

46. *If yes to 27* During the past month, how often did your **X** year-old eat **chocolate or any other types of candy**?
    - ☐ 1 time last month
    - ☐ 2-3 times last month
    - ☐ 1 time per week
    - ☐ 2 times per week
    - ☐ 3-4 times per week
    - ☐ 5-6 times per week
    - ☐ 1 time per day
    - ☐ 2 or more times per day
    - ☐ Prefer not to answer

47. During the past month, did your **X** year-old eat **doughnuts, sweet rolls, Danish, muffins, pan dulce, or Pop-Tarts**? Do **not** include sugar-free items.
    - ☐ Yes
    - ☐ No
    - ☐ Don't know
    - ☐ Prefer not to answer

48. *If yes to 29* During the past month, how often did your **X** year-old eat **doughnuts, sweet rolls, Danish, muffins, pan dulce, or Pop-Tarts**?
    - ☐ 1 time last month
    - ☐ 2-3 times last month
    - ☐ 1 time per week
    - ☐ 2 times per week
    - ☐ 3-4 times per week
    - ☐ 5-6 times per week
    - ☐ 1 time per day
    - ☐ 2 or more times per day
    - ☐ Prefer not to answer

49. During the past month, did your **X** year-old eat **cookies, cake, pie or brownies**? Do **not** include sugar-free kinds.
    - ☐ Yes
    - ☐ No
    - ☐ Don't know
    - ☐ Prefer not to answer

50. *If yes to 31* During the past month, how often did your **X** year-old eat **cookies, cake, pie or brownies**?
    - ☐ 1 time last month
    - ☐ 2-3 times last month

- ☐ 1 time per week
- ☐ 2 times per week
- ☐ 3-4 times per week
- ☐ 5-6 times per week
- ☐ 1 time per day
- ☐ 2 or more times per day
- ☐ Prefer not to answer

51. During the past month, did your **X** year-old eat **ice cream or other frozen desserts**? Do **not** include sugar-free kinds.
    - ☐ Yes
    - ☐ No
    - ☐ Don't know
    - ☐ Prefer not to answer

52. *If yes to 33* During the past month, how often did your **X** year-old eat **ice cream or other frozen desserts**?
    - ☐ 1 time last month
    - ☐ 2-3 times last month
    - ☐ 1 time per week
    - ☐ 2 times per week
    - ☐ 3-4 times per week
    - ☐ 5-6 times per week
    - ☐ 1 time per day
    - ☐ 2 or more times per day
    - ☐ Prefer not to answer

53. During the past month, did your **X** year-old eat **fast food**? Examples include McDonald's, Burger King, Chick-fil-A, Dunkin' Donuts, Subway, and Taco Bell.
    - ☐ Yes
    - ☐ No
    - ☐ Don't know
    - ☐ Prefer not to answer

54. *If yes to 35* During the past month, how often did your **X** year-old eat **fast food**?
    - ☐ 1 time last month
    - ☐ 2-3 times last month
    - ☐ 1 time per week
    - ☐ 2 times per week
    - ☐ 3-4 times per week
    - ☐ 5-6 times per week
    - ☐ 1 time per day
    - ☐ 2 or more times per day
    - ☐ Prefer not to answer

If yes to 35 For the next set of questions, please think about the foods your **X** year-old eats at a fast food or counter service restaurant.

55. When you take your **X** year-old to a fast food or counter service restaurant, which **menu** do you order from for your child? Choose one.
    - ☐ Adult/regular menu
    - ☐ Kid's menu
    - ☐ Sometimes the adult menu, sometimes the kid's menu
    - ☐ Items from both
    - ☐ Prefer not to answer

56. When you take your **X** year-old to a fast food or counter service restaurant, what **side(s)** do you order for your child? Check all that apply.
    - ☐ French fries/fried potatoes
    - ☐ Fresh fruit (apple slices, fruit cup)
    - ☐ Apple sauce
    - ☐ Yogurt
    - ☐ Other: ________________
    - ☐ Prefer not to answer

57. When you take your **X** year-old to a fast food or counter service restaurant, what **beverage** do you order for your child? Check all that apply.
    - ☐ Water
    - ☐ Milk
    - ☐ Juice
    - ☐ Soda/coke/pop
    - ☐ Other: ________________
    - ☐ Prefer not to answer

58. How often do you order **dessert** for your **X** year-old at a fast food or counter service restaurant? Choose one.
    - ☐ Never
    - ☐ Occasionally
    - ☐ About half the time
    - ☐ More than half the time
    - ☐ Always
    - ☐ Prefer not to answer
